# Neutrophil Expression of T and B Immunomodulatory Molecules in HIV Infection

**DOI:** 10.3389/fimmu.2021.670966

**Published:** 2021-12-17

**Authors:** Mercedes Márquez-Coello, Cristina Ruiz-Sánchez, Andrés Martín-Aspas, Clotilde Fernández Gutiérrez Del Álamo, Francisco Illanes-Álvarez, Sara Cuesta-Sancho, José-Antonio Girón-González

**Affiliations:** ^1^ Unidad de Enfermedades Infecciosas, Servicio de Medicina Interna, Hospital Universitario Puerta del Mar, Facultad de Medicina, Universidad de Cádiz, Cádiz, Spain; ^2^ Instituto de Investigación e Innovación en Ciencias Biomédicas de Cádiz (INiBICA), Cádiz, Spain; ^3^ Servicio de Microbiología, Hospital Universitario Puerta del Mar, Facultad de Medicina, Universidad de Cádiz, Cádiz, Spain

**Keywords:** HIV, neutrophils, BAFF, APRIL, bacterial translocation, interleukins, arginase-1, PDL-1

## Abstract

**Objective:**

Evaluate the expression of B and T cell immunomodulatory molecules in polymorphonuclear neutrophils (PMN) in HIV-infected patients.

**Methods:**

HIV load, bacterial translocation and neutrophils’ expression of T [programmed death ligand, interleukin-10+, arginase 1+] and B [BAFF, APRIL] molecules were analyzed in different cohorts and time points: a control group of 25 healthy individuals and two groups of HIV-infected patients. Group 1 of patients included 35 untreated patients, studied at baseline and after antiretroviral therapy (ART). Group 2 was composed of 25 patients with undetectable viral load after a median of 101 months of ART prior to inclusion in the study.

**Results:**

Compared with the control group, group 1 patients showed increased bacterial translocation and their PMN had a significantly higher expression of T and B-cell immunomodulatory molecules, both at baseline and after 12 months of ART. Group 2 patients showed reduced bacterial translocation levels when compared with group 1 patients after 12 months of treatment. PMN expression of B-cell modulators was similar between group 2 patients and healthy controls, although the expression of T-cell modulators remained increased.

**Conclusion:**

In HIV-infected patients, the expression of B-cell stimulatory and T-cell suppressive molecules by neutrophils was increased at baseline and after a limited time of therapy. After a prolonged period of ART, only PMNs expression of T-cell immunosuppressive molecules remained elevated.

## Introduction

Polymorphonuclear neutrophils (PMNs) have been considered to be effector elements of the antimicrobial immune response (1). PMNs recognize microbes through pathogen-associated molecular patterns, including Toll-like receptors (TLRs). Activation of TLRs in PMNs induces the production of proinflammatory cytokines, expression of adhesion molecules and activation of reactive oxygen species (ROS) (2).

The influence of PMNs on adaptive immunity has gained relevance in recent years. In acute bacterial infection, PMNs synthesize interleukin (IL)-12, provoking T cell differentiation in T helper 1 (Th1) cells. Under other circumstances, such as during chronic viral infections, PMNs are able to suppress T-lymphocyte activation (3). The neutrophil-dependent T cell down-regulation is mediated by the synthesis of arginase-1 (4), IL-10 production (5), programmed death antigen-1 ligand (PDL-1) overexpression (6) and transformation in myeloid-derived suppressor cells (7).

PMNs also synthesize B lymphocyte activating factors, such as the B cell-activating factor of the tumor necrosis factor family (BAFF) and a proliferation-inducing ligand (APRIL) (8). After binding to their receptors on B cells, BAFF and APRIL promote the survival and differentiation of B cells amongst plasma cells, stimulating the synthesis of immunoglobulin M (IgM) and a switch to IgG or IgA (8). Microbes stimulate BAFF and APRIL expression in PMNs, macrophages and dendritic cells, either by binding to TLRs or indirectly *via* interferon I and II synthesis (9).

After acute infection by the human immunodeficiency virus (HIV), a massive depletion of CD4+ T cells in gut-associated lymphoid tissues occurs and persists throughout the chronic stage (10). The administration of antiretroviral therapy (ART) only partially repairs gut mucosal injury (10, 11). Due to the CD4+ T cell loss and intestinal barrier damage, microbial translocation occurs in HIV-infected patients (12) showing an increase in serum levels of lipopolysaccharide and 16S ribosomal DNA (16S rDNA), which are markers of this bacterial translocation (12).

It is theoretically possible that T or B-cell immunomodulatory molecules in PMNs could be induced in HIV-infected patients either by HIV itself, *via* TLR7/8 interaction (13), or by bacterial-derived products secondary to bacterial translocation, either *via* TLR4-bacterial lipopolysaccharide or TLR9-16S rDNA interactions (2). Attending to this hypothesis, only a limited information about expression of T-cell inhibitory molecules (arginase-1, PDL-1) by PMNs has been reached in HIV-infected individuals (14, 15). The analysis of the expression of B-cell immunomodulatory molecules by PMNs has not been performed.

The objective of this study is to perform a quantitative evaluation of the expression of T and B cell modulatory molecules in PMNs in HIV-infected patients a) before ART, a situation in which both stimuli, HIV itself and microbial-translocation-derived products, could be operative, b) after 12 months of treatment, when gut barrier damage and bacterial translocation persist but HIV replication is controlled, and c) in a sample of HIV-infected individuals that had undergone more than 8 years (101 months) of therapy, to evaluate the long-term consequences of viral suppression on the expression of these molecules in PMNs.

## Patients And Methods

### Study Design

A prospective, observational study of consecutive cases of HIV infection recruited from a cohort of patients followed up in an HIV outpatient clinic at the Puerta del Mar University Hospital (Cádiz, Spain) was conducted.

60 HIV-infected adults were divided in the following groups: 1) Group 1 included 35 naïve patients. They were analyzed at the time of inclusion in the study and after 6 and 12 months with ART. 2) Group 2 included 25 patients with chronic HIV infection and ART-induced undetectable HIV viral loads at inclusion [undetectable HIV loads for a median of 101 months (range, 60–154 months)]. Comparison of results of PMNs derived molecules between group 1 patients after 12 months of treatment (when undetectable HIV viral load had been obtained) and group 2 individuals (with a longer period of undetectability) was performed. Twenty-five age- and gender-matched healthy individuals were recruited from voluntary hospital workers as healthy controls.

The exclusion criteria were as follows: 1) clinical records suggesting acute HIV infection; 2) active or other opportunistic infections [including viral hepatitis, *Pneumocytis jirovecii*, toxoplasmosis, tuberculosis, cytomegalovirus infections, etc] or neoplasms. Our screening procedure followed the guidelines established by the Spanish Group for AIDS Study (www.gesida.seimc.org); 3) active drug use (cocaine, heroin, amphetamines) or significant alcohol ingestion (greater than 50 g/day); 4) treatments that could have modified the determination of inflammation-related molecules or cells (pentoxifylline, anti-inflammatory or immunosuppressive drugs); 5) red blood cell or plasma transfusion in the month before inclusion; and 6) antibiotic treatment, which might modify the intestinal flora.

HIV infection was diagnosed using an chemiluminescent microparticle immunoassay (Alinity HIV Ag/Ab Combo assay, Ludwigshafen, Germany) for the simultaneous qualitative detection of HIV p24 antigen and antibodies to HIV type 1 and type 2 in serum. Positive results were confirmed by detection of HIV load (Abbott RealTime HIV-1, Abbott Park, IL, USA).

The duration of the HIV infection was established based on the first positive anti-HIV test.

When the HIV load was less than 50 copies/ml (Abbott RealTime HIV-1, Abbott Park, IL, USA), HIV replication was considered to be controlled.

Increased serum concentration of intestinal fatty acid-binding protein (I-FABP) was indicative of gut barrier disruption (16). The bacterial translocation was detected by serum 16S rDNA levels (17).

### Study Schedule

The protocol included: 1) clinical history, nadir CD4+ T cell count, and time with undetectable HIV load; 2) CD4+ T cell count, CD4+/CD8+ T cells ratio, and HIV RNA levels at inclusion in the study; 3) blood count, biochemistry, and coagulation determinations by automated procedures; 4) peripheral blood sampling at inclusion for analysis of bacterial translocation, immune and inflammatory parameters.

In patients with detectable HIV loads at baseline (Group 1), ART was initiated and followed for 12 months according to the Spanish Group for AIDS Study guidelines [https://gesida-seimc.org]. Their immune parameters were analyzed every six months.

Current ART was continued in those patients with undetectable HIV loads at inclusion (group 2) and we only determined their immune parameters at inclusion.

### Laboratory Methods

#### Sample Collection

Serum was obtained by centrifugation at 1600 g during 15 min at room temperature from blood samples in pyrogen-free heparinized tubes (Biofreeze, Costar, USA) and subsequently frozen down at -80°C until its use.

#### 16S rDNA Quantification

16S rDNA was detected after an initial extraction of DNA (QIAamp DNA Mini Kit; QIAgen, Hilden, Germany) and quantification by spectrophotometry (BioRad, Hercules, CA, USA). 16S rDNA region of *E. coli* was amplified using the following primers: 16S R 5´-ACC-GCC-ACT-GCT-GCT-GGC-AC-3´ and 16S F 5´-AGA-GTT-TGA-TCA-TGG-CTG-AG-3’ (IDT, Coralville, Iowa, USA). A polymerase chain reaction (PCR) was performed with a standard curve using an *E. coli* colony for quantification in a CFX Connect Real-Time PCR System (CFX Connect Real-Time PCR System, Bio-Rad Laboratories, Inc. CA, USA). The standard curve of *E. coli* was obtained from a colony, provided by the Microbiology Service of the Puerta del Mar University Hospital. After heat shock (90°C for 10 min and 10°C for 10 min) and centrifugation, the supernatant was collected and the concentration was measured by spectrophotometry. After serial dilutions of the colony sample, the RT PCR was carried out together with 3 samples per group of patients/controls to verify that the concentrations of the latter are within the curve obtained. The entire process was carried out in a biological safety horizontal flow hood. An exclusive pipette set for 16S PCR and filter tips were used. RT PCR was carried out on 8-well strips. As a precaution, every strip was covered and stored at 4°C when loaded. Finally, the samples were centrifuged and run in CFX.

#### ELISAS

Quantikine Human Immunoassays (R&D, Minneapolis, MN, USA) were used to quantify serum concentrations of I-FABP, IL-6 and IL-10. Immunoturbidometry was used to analyze serum immunoglobulin concentrations.

#### Flow Cytometry

Fresh cells from whole lysed blood were fixed and stained with blue-fluorescent reactive dye (Live/Dead Fixable Dead Cell Stain Kit; Invitrogen, Carlsbad, CA, USA) to exclude dead cells from analysis. Then, cell suspensions were stained for surface markers, and subsequently, after permeabilization with the Intra Stain (Dako, Denmark A/S), for intracellular molecules. Mouse isotype control conjugates with PE, PerCP, FITC and APC were used to confirm the specificity of the staining and to discriminate the sample from the background.

Stained cells were washed, acquired and analyzed in a FACSCalibur cytometer, using CellQuest software (BD Bioscience, San Jose, CA, USA). In each case, 100000 cells were acquired.

Granulocyte populations were gated based on forward (FSC-H) and side (SSC-H) scatter. In this gate, CD15+ and CD14- cells were selected. For PMN characterization, anti-CD16 (clone VEP13, Miltenyi Biotec, Bergisch, Gladbach, Germany), anti-CD15 (clone W6D3, Becton Dickinson, San Jose, CA, USA), and anti-CD14 (clone TET2, Becton Dickinson) were used (**Supplementary Figure 1**).

Membrane expression of CD11b (clone ICRF44, Becton Dickinson) and PDL-1 (clone 29E.2A3, Becton Dickinson) were analyzed. Intracellular expression of molecules using anti-IL-6 (clone 1936, R&D), anti-IL-10 (clone JES3-9D7, Milteny Biotec), anti h/m arginase-1 (Polyclonal sheep IgG, R&D Systems, Minneapolis, USA), anti-human CD257 (BAFF, BLYS) (clone T7-241, Biolegend, San Diego, CA, USA) and anti-APRIL (clone A3D8, Biolegend) were determined (**Supplementary Figures 2**, **3**). PMNs molecule expression is indicated as median fluorescence intensity (MFI).

To avoid nonspecific staining and/or antibody binding to non-target intracellular proteins or neutrophil Fc receptors (18), the following actions were taken: 1) To ensure that the antibodies did not give false positive signals because of the cross-react with intracellular proteins or binding to non-specifically to FcγRII/CD32 and FcγIII/CD16, cells were treated with a FcR blocking reagent (Miltenyi Biotec). 2) In our protocol, a negative control was always run only with blood, but processed as if it had antibodies. Therefore, when results were analyzed, the signal in the tube with no antibodies was our negative control (cell autoflorescence).

Lymphocytes were gated based on forward (FSC-H) and side (SSC-H) scatter for determination of CD4+ (clone SK3) and CD8+ (clone SK1) populations. Anti CD19 (clone HIB 19, Becton Dickinson, San Jose, CA, USA), anti CD27 (clone M-T271, Becton Dickinson, San Jose, CA, USA) and anti IgD (clone IA6-2, Becton Dickinson, San Jose, CA, USA) were used for B lymphocytes.

### Statistical Analysis

Data were expressed as absolute numbers (percentage) or as median values (25-75 interquartile range (IQR). Categorical variables were compared using the chi-square test or Fisher’s exact test. The Mann-Whitney U test or ANOVA was used to compare quantitative variables from two independent groups. For comparison of the three or more independent groups, Kruskal-Wallis test was used. Friedman’ or Wilcoxon’s rank tests were used to perform paired analysis of variables. The association between quantitative variables was analyzed using the Spearman correlation test. A two-tailed p value of <0.05 was considered to be significant. SPSS 22.0 statistical software package (SPSS Inc., Chicago, IL, USA) was used to perform statistical analysis.

### Ethical Aspects

This study was performed according to the Helsinki Declaration. The ethical research committee of Hospital Puerta del Mar (Cádiz, Spain) approved the project. Each participant gave written informed consent.

## Results

The haematological, immune and virological characteristics of untreated patients (Group 1) and controls are shown in **Table 1**. 71% of those patients (n=25) showed a CD4+ T cell count lower than 500/mm^3^.

**Table 1:** Hematological, immune and virological characteristics and gut barrier lesion and bacterial translocation markers of healthy controls and HIV-infected patients.

	Healthy controls (n = 25)	Chronic HIV-infected patients with detectable HIV load at baseline (Group 1) (n=35)		Chronic HIV-infected patients with suppressed HIV load at inclusion (Group 2) (n = 25).	p 1 vs 2	p 1 vs 3	p 1 vs 4	p 2 vs 3	p 3 vs 4
		Prior to antiretroviral therapy	After 12 months of antiretroviral therapy						
	(1)	(2)	(3)	(4)					
Age (years)	36 (26-42)	39 (28-47)	40 (29-48)	41 (28-49)	0.641		0.234		0.948
Sex male (n,%)	20 (80)	28 (80)	28 (80)	18 (72)	1.000		0.742		0.543
Time from HIV diagnosis (months)		6 (1-28)	18 (13-40)	101 (60–154)					<0.001
**Haematological variables**									
Leukocytes/mm^3^	5885 (4620-7283)	5720 (5245-7385)	5860 (5058-6823)	5740 (4905 – 6750)	0.755	0.989	0.897	0.918	1.000
PMN/mm^3^	3125 (2520-3795)	2860 (2230-4115)	2955 (2563-3845)	2700 (2490 – 3625)	0.685	0.838	0.589	0.501	0.941
Monocytes/mm^3^	520 (365-620)	560 (440-740)	540 (435-643)	500 (390 – 700)	0.062	0.486	0.412	0.551	0.824
Lymphocytes/mm^3^	1905 (1528-2655)	2130 (1395-2675)	1885 (1138-2500)	1820 (1605 – 2535)	0.742	0.539	0.912	0.063	0.706
**Immune-virological variables**									
CD4+ T cell/mm^3^ at diagnosis		386 (158-636)	386 (158-636)	435 (148–607)					0.642
CD4+ T cells/mm^3^	694 (625-1352)	302 (153 – 622)	433 (292–772)	707 (600–905)	<0.001	<0.001	0.746	0.001	0.013
CD8+ T cells/mm^3^	506 (340 – 643)	1051 (642 - 1528)	691 (490-1030)	842 (549 – 1107)	<0.001	0.013	<0.001	0.088	0.067
CD4/CD8 ratio	1.76 (1.65 – 2.07)	0.25 (0.13 – 0.49)	0.56 (0.43-0.77)	0.96 (0.63 – 1.30)	<0.001	<0.001	<0.001	<0.001	0.037
HIV load (copies/ml) at inclusion		13958 (1926 – 130978)	< 50 (<50 - <50)	<50 (<50 – <50)				<0.001	1.000

HIV, Human immunodeficiency virus; PMN, Polymorphonuclear.

Data are provided as absolute number (percentage) or as median (interquartile range).

Serum I-FABP and 16S rDNA concentrations were significantly higher in untreated patients than in controls (**Table 2**), and a significant correlation between I-FABP concentration and 16S rDNA levels (r=0.306, p=0.026) was detected.

**Table 2:** Gut barrier status, bacterial translocation and immune characteristics of healthy controls, patients with chronic infection, untreated at baseline, analyzed after 12 months of antiretroviral therapy, and of chronically treated HIV patients with undetectable HIV load at inclusion.

	Healthy controls (n = 25)	Chronic HIV-infected patients with detectable HIV load at baseline (Group 1) (n=35)		Chronic HIV-infected patients with suppressed HIV load at inclusion (Group 2) (n = 25).	p 1 vs 2	p 1 vs 3	p 1 vs 4	p 2 vs 3	p 3 vs 4
		Prior to antiretroviral therapy	After 12 months of antiretroviral therapy						
	(1)	(2)	(3)	(4)					
**I-FABP (ng/ml)**	2.3 (1.4–2.8)	3.7 (2.4-6.3)	5.1 (4.2-8.1)	6.3 (4.0–8.6)	<0,001	<0,001	<0,001	0.105	0,838
**16S rDNA (copies/ml)**	276 (164–448)	1713 (1085-3291)	1476 (900–2692)	1257 (832–1582)	<0.001	<0.001	0.001	0.469	0.037
**Serum IL-6 (pg/ml)**	5 (3–7)	11 (6-17)	26 (18-50)	11 (7–18)	<0.001	<0.001	<0.001	<0.001	<0.001
**Serum IL-10 (pg/ml)**	14 (8-26)	26 (18-50)	55 (19-65)	23 (11-40)	<0.001	<0.001	0.047	0.002	0.012
**PMN-CD11b+ (MFI)**	25 (18 - 61)	51 (27-95)	61 (19 – 86)	42 (22 – 93)	0.019	0.003	0.027	0.535	0.113
**PMN-IL6+ (MFI)**	86 (66 – 187)	275 (166-645)	636 (454 – 740)	223 (135 – 623)	<0.001	<0.001	0.002	0.003	0.017
**PMN-PDL-1+ (MFI)**	113 (80 – 154)	171 (80-395)	401 (358 – 497)	96 (57 – 350)	0.019	<0.001	0.884	<0.001	<0.001
**PMN-IL10+ (MFI)**	40 (22 – 56)	68 (31-165)	193 (143 – 208)	54 (32 – 158)	0.004	<0.001	0.019	<0.001	<0.001
**PMN-Arginase 1+ (MFI)**	45 (23 – 655)	794 (375-1234)	1120 (840 – 1428)	632 (167 – 1228)	0.001	<0.001	0.006	0.002	0.039
**PMN-BAFF+ (MFI)**	87 (78 – 134)	139 (72-358)	371 (315 – 408)	82 (46 – 247)	0.020	<0.001	0.651	0.039	<0.001
**PMN-APRIL+ (MFI)**	105 (69 – 129)	154 (86-291)	393 (336 – 443)	74 (43 – 246)	0.039	<0.001	0.503	<0.001	<0.001
**B cells/mm3**	163 (122-202)	135 (65-228)	157 (80-265)	168 (117-247)	0.090	0.594	0.821	0.104	0.613
**Serum Ig G (mg/dl)**	1018 (822-1065)	1292 (933 – 1651)	1174 (1028-1366)	1088 (929-1282)	<0.001	0.001	0.121	0.758	0.019
**Serum Ig A (mg/dl)**	197 (145-261)	310 (226 – 426)	271 (205-361)	241 (160-291)	<0.001	0.003	0.089	0.163	0.047
**Serum Ig M (mg/dl)**	115 (86-137)	105 (75 – 160)	99 (73-141)	95 (65-122)	0.160	0.439	0.126	0.906	0.344

HIV, Human immunodeficiency virus; I-FABP, Intestinal fatty acid binding protein; IL-6, interleukin 6; IL-10, Interleukin 10; PMN, Polymorphonuclear;

MFI, median fluorescence intensity; Ig, Immunoglobulin.

Data are provided as median (interquartile range).

Serum IL-6 and IL-10 concentrations were significantly higher in untreated HIV-infected individuals than in controls (**Table 2**).

### Activation Level of PMNs in Untreated HIV-Infected Patients

The activation level of PMNs was measured based on the membrane expression of CD11b and the intracellular expression of IL-6. The MFI of CD11b and intracytoplasmic IL-6 were significantly increased in Group 1 patients (**Table 2** and **Supplementary Figure 2**).

### PMNs Expression of T and B Cell Modulatory Molecules in Untreated HIV-Infected Patients

Membrane expression of PDL-1 and intracytoplasmic expression of IL-10 and arginase-1 as T cell modulatory molecules in PMNs were analyzed, showing an increase in untreated HIV-infected patients (**Table 2** and **Supplementary Figure 2**).

The intracellular expression of BAFF and APRIL in PMNs was significantly higher in untreated HIV-infected individuals (**Table 2** and **Supplementary Figure 3**).

No significant differences between activation parameters or expression of T or B modulatory molecules in PMNs were observed between patients with more or less than 500 T CD4+ cells/mm^3^ (data not shown).

### Evolution of Gut Barrier Alteration, Bacterial Translocation and Immune Markers in Group 1 Patients After ART

All Group 1 patients started ART and achieved undetectable HIV loads at 6 months. Patients were treated either with tenofovir alafenamide and emtricitabine (31 cases) or abacavir and lamivudine (4 cases) associated with integrase inhibitors (23 cases), darunavir/cobicistat (7 individuals) or rilpivirine (5 patients). A significant increase in the CD4+ T cell count in patients was observed (**Table 1**). No significant differences were found in B cell counts or IgG, IgA or IgM serum concentrations between data collected at baseline and after 12 months of therapy (**Table 2**).

During the follow-up, I-FABP concentrations remained elevated, without significant differences when compared with baseline values (**Table 2**). Likewise, 16S rDNA continued to be elevated despite ART (**Table 2**).

**Figure 1:**
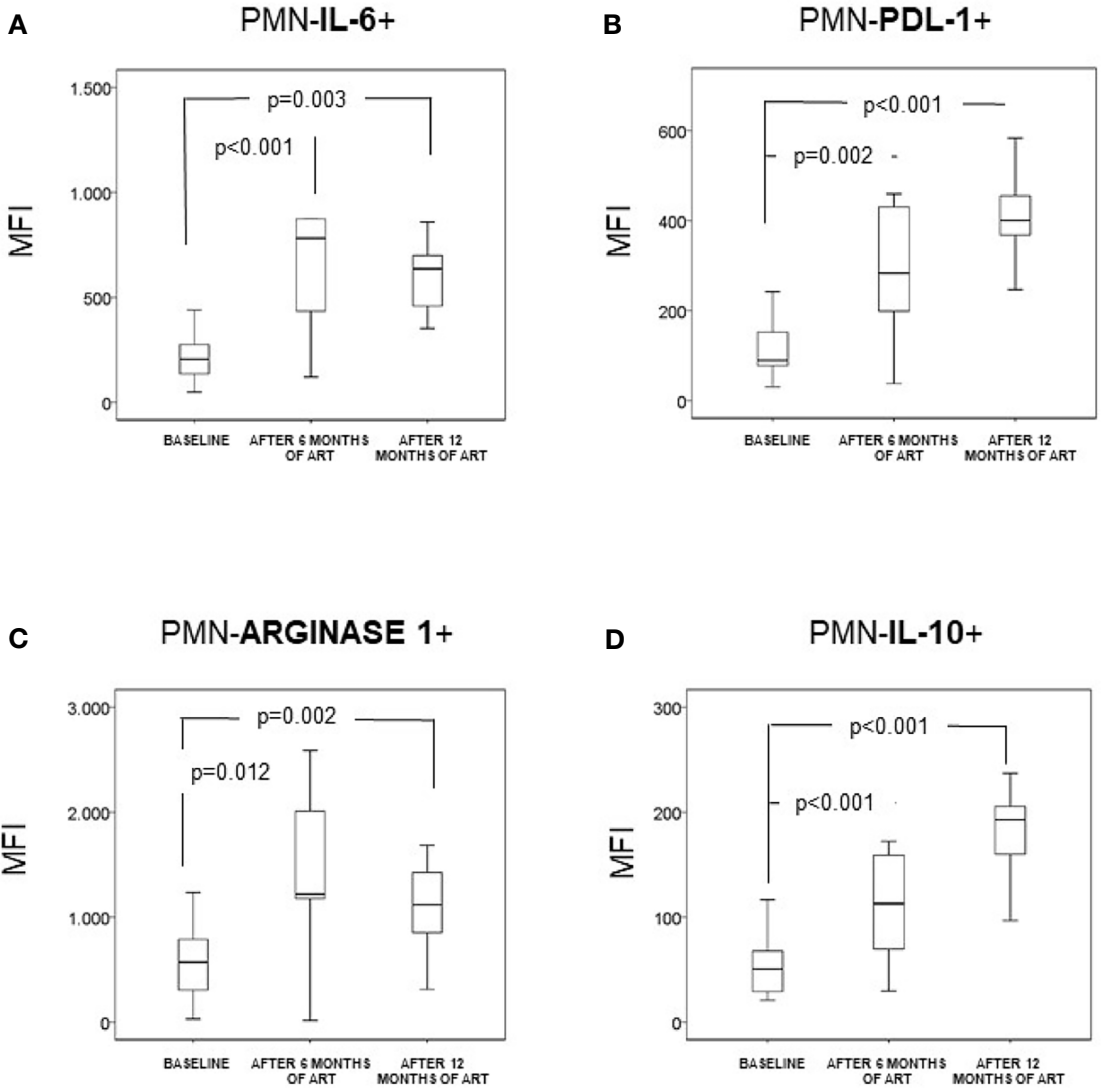
Neutrophils (CD14-CD15+CD16+) (PMN) expression of intracellular IL-6 **(A)**, intracellular arginase 1 **(B)**, membrane PDL-1 **(C)**, and intracellular IL-10 **(D)** in naïve HIV-infected patients (n = 35). They were analyzed at baseline and after 6 and 12 months of antiretroviral treatment. Data are provided as median, interquartile range (boxes) and range of the mean fluorescence intensity (MFI), at baseline and after 6 and 12 months of antiretroviral therapy.

**Figure 2:**
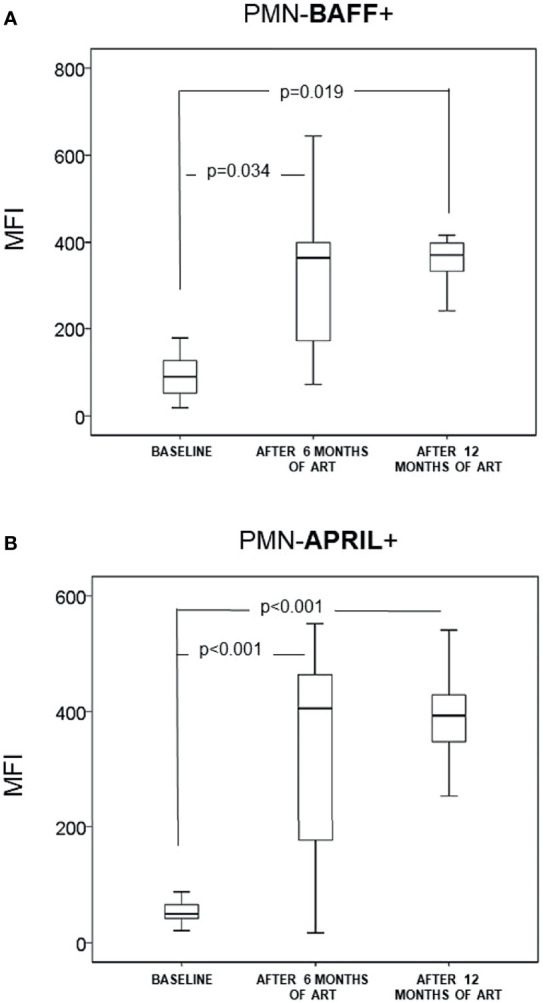
Neutrophils (CD14-CD15+CD16+) (PMN) expression of B cell-modulating markers: intracellular BAFF **(A)** and intracellular APRIL **(B)** in naïve infected patients (n = 35). They were analyzed at baseline and after 6 and 12 months of antiretroviral treatment. Data are provided as median, interquartile range (boxes) and range of the mean fluorescence intensity (MFI), at baseline and after 6 and 12 months of antiretroviral therapy.

The MFI of IL-6 and T (PDL-1, Arginase-1, IL-10) and B (BAFF and APRIL) modulatory molecules expressed in PMNs had increased significantly after 6-month of ART when compared with the baseline values. Differences between values of MFI obtained after 6 and 12 months of follow-up were non-significant (**Figures 1** and **2**).

IL-6 serum concentrations increased significantly during the follow-up [baseline, 11 (6–17) pg/ml; after 6 months of ART, 25 (16-34) pg/ml, p<0.001 when compared with baseline; after 12 months of ART, 26 (18-50) pg/ml, p<0.001 when compared with baseline; p=0.270 when compared with 6 months of ART]. Also, IL-10 serum concentrations increased significantly during the follow-up [baseline, 25 (18-50) pg/ml; after 6 months of ART, 55 (38-69) pg/ml, p=0.001 when compared with baseline; after 12 months of ART, 55 (19-65) pg/ml, p=0.002 when compared with baseline; p=0.100 when compared with 6 months of ART].

### Comparison of PMNs Expression of T- and B-Immunomodulatory Molecules in Naïve HIV-Infected Patients (Group 1) After ART During 12 Months and Patients With Chronically Undetectable HIV Load (Group 2)

Characteristics observed after 12 months of ART in the 35 HIV-infected individuals that had been untreated at baseline (Group 1) were compared with those of 25 chronically infected patients with undetectable HIV at inclusion as a consequence of previous ART (Group 2) [undetectable HIV load for a median of 101 (range, 41–317) months] (**Table 1**). Group 2 individuals had been treated either with tenofovir alafenamide and emtricitabine (21 patients) or abacavir and lamivudine (4 patients) combined with integrase inhibitors (18 patients), darunavir/cobicistat (6 patients) or rilpivirine (1 patient). A significantly lower 16S rDNA level and serum concentration of IL-6 and IL-10, although not of I-FABP, was observed in Group 2 patients when compared to group 1 individuals treated for only 12 months; however, serum I-FABP, 16S rDNA, IL-6 and IL-10 levels were significantly higher than those of healthy controls (**Table 2**).

The MFI of IL-10 and arginase-1 in peripheral blood PMNs was significantly lower in Group 2 than in Group 1 patients treated for 12 months, although it was higher than in healthy controls (**Table 2**).

The MFI of BAFF and APRIL in PMNs was significantly lower in chronically infected patients with undetectable HIV at inclusion (Group 2) compared with those treated for 12 months (Group 1). MFI of BAFF and APRIL in PMNs of Group 2 individuals were similar to those observed in healthy controls (**Table 2**).

B lymphocyte/mm^3^ counts were similar in healthy controls, Group 1 patients after 12 months of ART and Group 2 patients. Serum concentrations of IgG and IgA continued increased in Group 1 patients when compared with healthy controls. In contrast, IgG, IgA and IgM levels were similar in Group 2 HIV-infected patients and healthy controls (**Table 2**).

## Discussion

Results of this work support the fact of the increased PMN lymphocyte expression of B lymphocyte markers BAFF and APRIL and T lymphocyte molecules PDL-1, IL-10 and arginase-1 in naïve HIV-infected patients. These cell modifications persist after 12 months of ART-induced HIV control, but only those related with T cell function continue to be present after a prolonged period of HIV undetectability.

PMNs from HIV-infected patients are activated. After recognition of infectious antigens *via* TLRs, PMNs trigger the synthesis of proinflammatory cytokines and the expression of adhesion molecules, such as CD11b (2). These data explain the increased expression of membrane CD11b and intracytoplasmic IL-6 in the PMNs of the HIV-infected patients that we analyzed in this study, thus supporting previous data (19).

Our data demonstrate that PMNs could influence adaptive immunity. In chronic viral infections, PMNs are able to suppress T-lymphocyte activation (3). The down-regulation of T cells by PMNs has been observed in HIV infection. This could be due to secretion of arginase-1, which depletes L-arginine from the medium and decreases the T cells viability (14), or through PDL-1 increased expression, which has the ability to interact with PD-1 expressed by T cells, and induces lymphocyte apoptosis (15). The expression of these molecules, as well as of the immunoregulatory IL-10 (20), was significantly increased in the PMNs of untreated HIV-infected patients in our series. This could be, in part, responsible for T cell dysfunction in HIV-infected individuals.

The IL-10 production by PMNs has been previously discussed in literature. Some authors have concluded that human neutrophils are unable to produce IL-10 (21), while others support the PMN capability to produce it (22–24). Our data are in agreement with those last authors. However, the heterogeneity of human neutrophils has been recognized (25) and thus both interpretations could be correct.

More interestingly, our data imply that PMNs are involved in modulating humoral immunity in these individuals. PMNs-derived BAFF and APRIL are implicated in the survival and differentiation of B cells, and in stimulating IgM synthesis and switching to IgG or IgA (8, 9). We observed an increase in the expression of BAFF and APRIL in untreated HIV-infected patients.

HIV itself, *via* TLR7/8 (13) interactions, or microbial derived products, *via* lipopolysaccharide-TLR4 or 16S rDNA-TLR9 interactions (2) could be stimuli implicated in these PMN responses. After ART treatment and controlled viral replication for at least 6 months, microbial translocation-derived molecules could be the main modulators of immune populations. Indeed, and consistent with previous data (10, 11), increases in I-FABP and 16S rDNA levels persist after ART.

The control of HIV replication is not achieved immediately after beginning ART, continuing the stimuli until undetectable HIV load is obtained. Thus, in our study, the data in which the HIV is eliminated are those patients 6 months after therapy. This could justify that serum IL-6 and IL-10 levels increase and the PMNs activation status and PMNs’ expression of T and B cell modulatory molecules continue to be elevated, even to a higher degree in patients after 6 months of ART, reaching a plateau between 6 and 12 months, after undetectable HIV loads have been reached. These findings support that the PMN modulation could be caused, among others, by the persistent intestinal microbial translocation. Viral persistence out of peripheral blood, treatment toxicity and other chronic-related factors may also be implicated in PMN modifications.

On the other hand, bacterial translocation could be influenced by intestinal microbioma, which has been detected to be different in untreated HIV-infected patients, with a depletion of butyrate-producing bacteria (*Lachnospiraceae* and *Ruminococcaceae* families) and a higher load of species prone to microbial translation (mainly *Enterobacteriaceae*) (26). Intestinal dysbiosis may result in increased neutrophil recruitment in intestinal mucosa, as a possible mechanism implicated in microbial translocation (27). Modification of gut microbiota, with a diminution of the number of bacteria with ability to translocate, could decrease the immune activation state in ART-treated HIV-infected individuals, as has been demonstrated by Serrano-Villar et al., using fecal microbial transplantation from healthy individuals (28). These aspects have not been analyzed in our article.

The observed PMN-induced B-cell stimulant response is similar to that detected in the splenic marginal zone (29). It has been theorized that, in the absence of disease, small quantities of intestinal microbial antigens gain access to systemic circulation, activate splenic sinusoidal endothelial cells and induce chemotaxis of PMNs. PMNs are reprogramed to eliminate antigens and provide positive B-cell (BAFF and APRIL expression) signals (9, 30, 31). In chronic inflammation states, an excessive number of PMNs with these phenotypical characteristics are observed in the spleen, and they can blur the limits of the splenic marginal zone and be observed in peripheral blood (32).

To assess the persistence of these changes in the PMNs, we included a sample of chronic HIV-infected patients treated with ART and presenting HIV undetectability for 101 months (Group 2). It is worthy of note that 16S rDNA levels were lower in these patients than in those treated for only 12 months (Group 1), suggesting a partial improvement in microbial translocation (10, 11). Even after more than 8 years of HIV undetectability, the expression of T-cell modulatory molecules (intracellular IL-10 and arginase-1), and serum IL-10 levels, continued to be elevated when compared with healthy controls. In contrast, and coincident with the decrease of 16S rDNA levels, B cell modulatory molecules levels in Group 2 individuals were significantly lower when compared with Group 1 patients treated for only 12 months. Furthermore, expression values of BAFF and APRIL by PMNs in Group 2 patients were similar to the values expressed by healthy controls. The differences in the T and B lymphocyte modulation by PMNs, depending on the length of the patients’ HIV undetectability (either 1 or 8 years), could be the result of the different sensitivities of PMN-derived T and B modulation molecules to the levels of 16S rDNA.

Serum immunoglobulins levels are influenced by B cell functional state. Indeed, hyperimmunoglobulinemia secondary to polyclonal activation represents an important finding of B cell dysfunction in HIV-infected patients (33–35). As in our study, other authors have reported that abnormally elevated levels of immunoglobulins persist after 12 months of HIV suppression (36, 37). Asides the T-cell influence on B-cell activation and intrinsic abnormalities of B cells (33, 34), the present article supports the additional influence of PMNs on B cell function in HIV-infected individuals and the ability of a prolonged period of HIV-replication control to normalize immunoglobulins concentration.

The possibility of non-specific binding to intracellular proteins could be a potential limitation of this work. However, we assume that the use of FcR blocking reagents, the negative controls to avoid the autofluorescence issue, the strict follow-up of the manufacturers’ recommendations and the acquisition of at least 100000 cells in each experiment support the validity of our measurements.

In conclusion, new findings about the pathogenesis of HIV infection are presented: 1) PMNs expression of T-cell regulatory molecules IL-10, PDL-1 and arginase-1 is increased in naïve HIV-infected patients and persists even when a prolonged HIV undetectability period is achieved. 2) PMNs expression of B-cell regulatory molecules BAFF and APRIL is increased in naïve HIV-infected patients and persists during a short period (12 months) of HIV undetectability, but normalizes after a more prolonged period (8 years), coinciding with the relative decrease of intestinal microbial translocation. 3) Bacterial translocation persists even after a prolonged period of undetectability. These findings suggest a possible contribution of bacterial translocation on B and T cell immunomodulation of PMNs.

## Data Availability Statement

The original contributions presented in the study are included in the article/supplementary files, further inquiries can be directed to the corresponding author.

## Ethics Statement

The studies involving human participants were reviewed and approved by Ethical Research Committee of Hospital Puerta del Mar (Cádiz, Spain). The patients/participants provided their written informed consent to participate in this study.

## Author Contributions

Conceived and designed the experiments: MM-C and J-AG-G. Performed the experiments: MM-C, CR, AM-A, CF, SC-S, and J-AG-G. Analyzed the data: MM-C, SC-S, and J-AG-G. Contributed reagents/materials/analysis tools: MM-C, CR, CF; FI-Á; SC-S, and J-AG-G. Wrote the draft: MM-C, SC-S, and J-AG-G. All authors contributed to conception of the study, and critical revision of the manuscript, and saw and approved the final version.

## Funding

This work was supported by the Instituto de Salud Carlos III, Acción Estratégica en Salud 2014 (No PI14/01779), Spain. Co-financed by FEDER (Fondo Europeo de Desarrollo Regional).

## Conflict of Interest

The authors declare that the research was conducted in the absence of any commercial or financial relationships that could be construed as a potential conflict of interest.

## Publisher’s Note

All claims expressed in this article are solely those of the authors and do not necessarily represent those of their affiliated organizations, or those of the publisher, the editors and the reviewers. Any product that may be evaluated in this article, or claim that may be made by its manufacturer, is not guaranteed or endorsed by the publisher.
