# Supplementary material for: Neutrophil Expression of T and B Immunomodulatory Molecules in HIV Infection
Source: Front Immunol. 2021 Dec 17;12:670966. doi: 10.3389/fimmu.2021.670966 (PMC8718872; doi:10.3389/fimmu.2021.670966)
Supplement: Supplementary file 1 [file DataSheet_1.pdf]

SUPPLEMENTARY MATERIAL

TITLE: Neutrophil expression of T and B immunomodulatory molecules in HIV infection

SHORT TITLE: Neutrophils in HIV infection

AUTHORS: Mercedes Márquez-Coello [1], Cristina Ruiz Sánchez [1], Andrés Martín-Aspas [1], Clotilde Fernández Gutiérrez Del Álamo [2], Francisco Illanes-Álvarez [1], Sara Cuesta-Sancho [1 †], José-Antonio Girón-González [1 †].

[1]. Unidad de Enfermedades Infecciosas, Servicio de Medicina Interna, Hospital Universitario Puerta del Mar, Facultad de Medicina, Universidad de Cádiz, Cádiz, Spain. Instituto de Investigación e Innovación en Ciencias Biomédicas de Cádiz (INiBICA)

[2] Servicio de Microbiología, Hospital Universitario Puerta del Mar, Facultad de Medicina, Universidad de Cádiz, Cádiz, Spain. Instituto de Investigación e Innovación en Ciencias Biomédicas de Cádiz (INiBICA)

[†] These authors have contributed equally to this work and share last authorship.

Supplementary figures

FIGURE LEGENDS

Supplementary FIGURE 1. **Gating strategy**. Gating strategy of polymorphonuclear neutrophils (PMN) based on (A) forward (FSC-H) and side (SSC-H) scatter, the gate is the PMN. A dot plot was made and CD15+ (FL3) and CD14- (FL4) cells were selected without (B) and with antibodies (C). Then, PMNs were characterized by anti-CD16 (FL1). They have been shown without and with antibody in (D) and (E) graphs. An overlay was made to visualize both populations (F).

Supplementary FIGURE 2**. Representative figure of polymorphonuclear (PMN) expression of IL-6 and T cell-modulating markers in a healthy individual and HIV infected patient.** PMNs were gated based on forward (FSC-H) and side (SSC-H) scatter for determination of granulocyte populations. In this gate, CD15+CD16+ cells were selected. (A) Anti-human IL-6 phycoerythrin conjugated (PE). (B) Anti-human arginase-1 fluorescent isocyanate conjugated (FITC). (C) Anti-human PDL-1 PE conjugated. (D) Anti-human IL-10 PE conjugated.

Supplementary FIGURE 3. **Representative figure of polymorphonuclear (PMN) expression of B cell-modulating markers in a healthy individual and HIV patient.** PMNs were gated based on forward (FSC-H) and side (SSC-H) scatter for determination of granulocyte populations. In this gate, CD15+CD16+ cells were selected. Markers are plotted on the X-axis of the histogram. (A) Anti-human BAFF phycoerythrin conjugated (PE). (B) Anti-human APRIL phycoerythrin conjugated (PE) conjugated.
