# Supplementary figures and images for: Neutrophil Expression of T and B Immunomodulatory Molecules in HIV Infection
Source: Front Immunol. 2021 Dec 17;12:670966. doi: 10.3389/fimmu.2021.670966 (PMC8718872; doi:10.3389/fimmu.2021.670966)

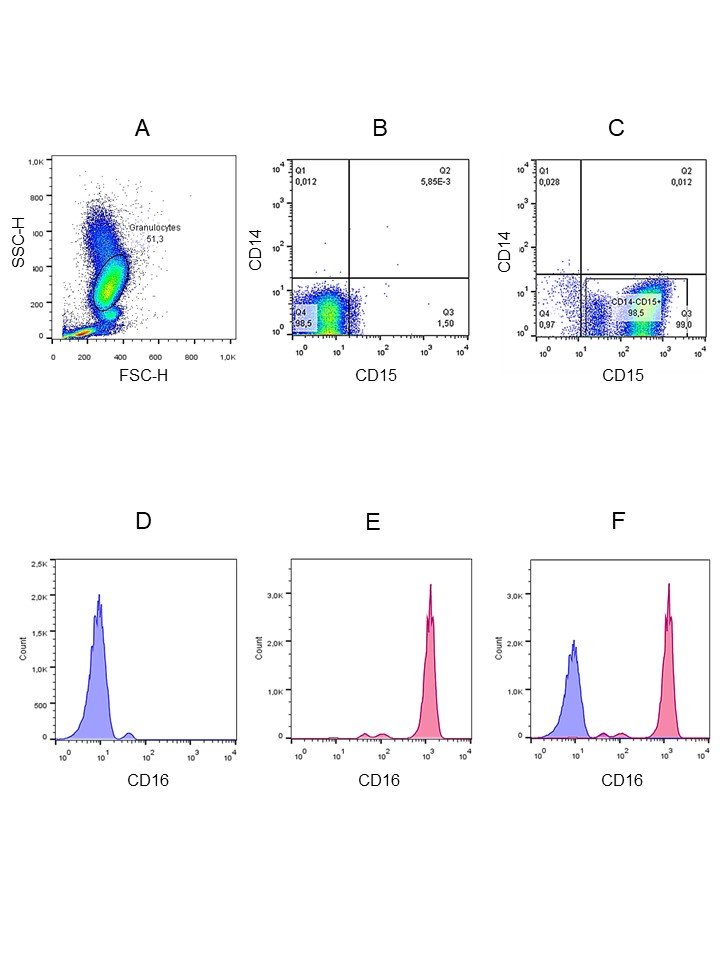

Supplement: Supplementary file 2 [file Image_1.jpeg]

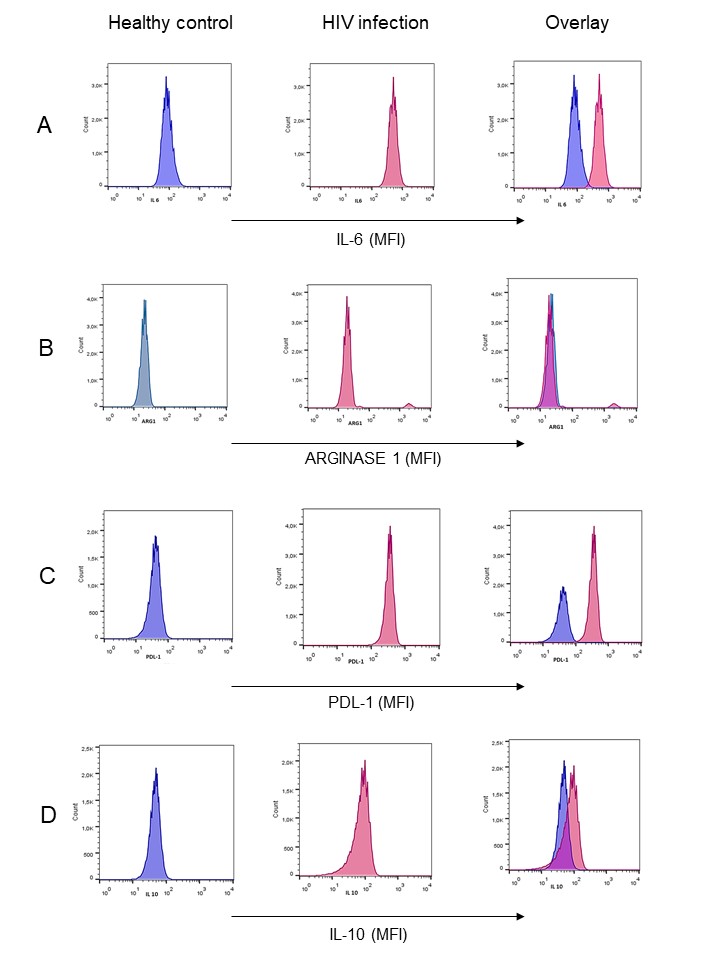

Supplement: Supplementary file 3 [file Image_2.jpeg]

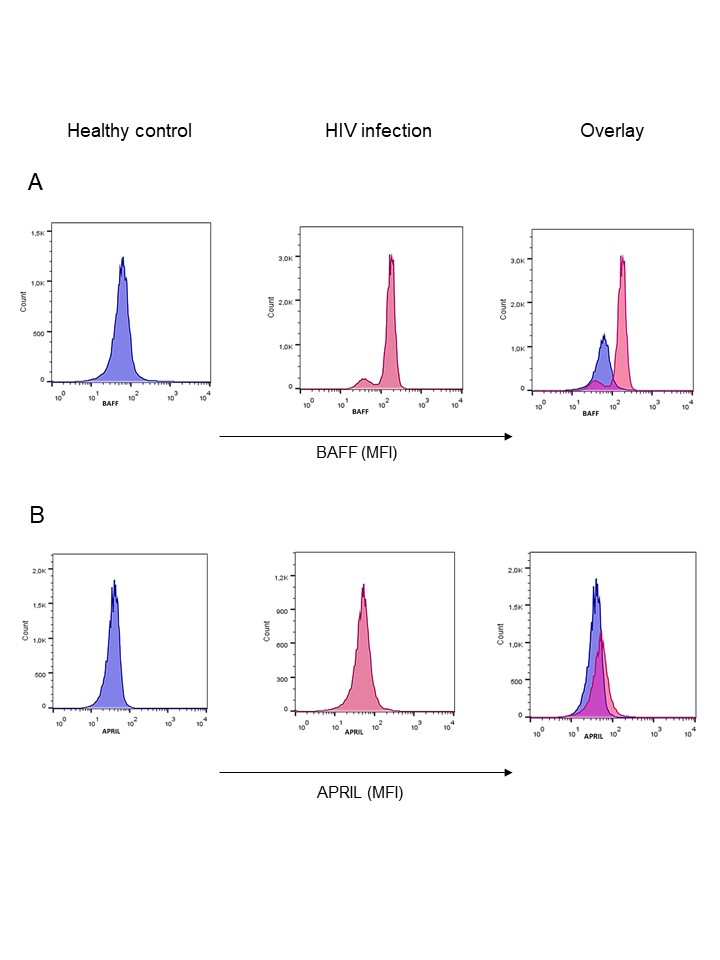

Supplement: Supplementary file 4 [file Image_3.jpeg]
